# Caveolae as a target to quench autoinduction of the metastatic phenotype in lung cancer

**DOI:** 10.1007/s00432-015-2074-3

**Published:** 2015-11-16

**Authors:** David John Garnett

**Affiliations:** Institute of Science Technology in Medicine, Keele University, Keele, Staffordshire ST5 5BG UK

**Keywords:** Caveolae, Metastasis, Gene expression, Proadifen, Statins

## Abstract

**Purpose:**

Mevalonate pathway inhibitors are potentially useful chemotherapeutic agents showing growth inhibition and pro-apoptotic effects in cancer cells. The effects of statins and bisphosphonates on cancer growth are attributed to a reduction in protein isoprenylation. Post-translational modification and activation of GTPase binding Ras superfamily permit the recruitment of these signal proteins to membranes where they mediate the cancer phenotype. Here, the effects of three inhibitors of the mevalonate pathway and one specific inhibitor of sterol regulatory element-binding proteins were studied in both an ER-negative, Ras-inactive breast (MDA-MB-231) and lung adenocarcinoma (CaLu-1) cells in vitro.

**Methods:**

Treated cells were subject to genome-wide gene expression profiling. A gene subset was established so that the epithelial to mesenchymal transition (EMT) could be observed and compared with signalling protein shifts.

**Results:**

Within the subset, some genes normally up-regulated during EMT were asymmetrically reduced by a Δ-24 DHCR inhibitor in the lung cells. Signalling proteins associated with caveolae were down-regulated by this oxidoreductase inhibitor, while those associated with membrane rafts were up-regulated.

**Conclusions:**

This study decouples isoprenylation effects from cholesterol events per se. The data support a hypothesis that caveolae are abolished by Δ-24 DHCR intervention and it is revealed that these microdomains are vital EMT signalling structures for lung cells but not ER- and Ras-negative breast cells. When signalling by extracellular signals is quenched by removal of the hydrophilic conduit provided by caveolae, the transcriptome responds by moving the cellular identity towards quiescence.

**Electronic supplementary material:**

The online version of this article (doi:10.1007/s00432-015-2074-3) contains supplementary material, which is available to authorized users.

## Introduction

The dissemination of solid tumour cell clones often precedes the detection of the primary cancer growth. The metastatic cascade is a multistep process beginning with infiltration through the organ of the primary tumour and into the blood stream (Talmadge and Fidler 2010). This stage has often occurred prior to first treatment [for example, 40 % of newly diagnosed patients with non-small lung cancer already have metastases (Goldstraw et al. 2007)], and it is therefore later steps of the cascade that represent opportunities for useful chemotherapeutic intervention: specifically suppression of existing disseminated cells at a pre-aggressive stage (Weber 2013). Typical anticancer drugs target replication and promote apoptosis, but these are ineffective strategies to kill latent clones and progress in this field has been impeded by a lack of validated predictive biomarkers for metastatic potential (Vignot et al. 2012). Angiogenesis has been successfully targeted (Shojaei 2012) by drugs such as bevacizumab, but newer therapies targeting other phenotypic changes or intrinsic genetic or epigenetic markers are sought to combat metastasis (Lehembre and Regenass 2012). Cholesterol pathway inhibitors are often incidental inhibitors of the isoprenoid pathway and have been studied in the context of cell cycle arrest and apoptosis (Mo and Elson 2004), but their putative effects on the epithelial–mesenchymal transition (EMT) and metastasis per se have not been fully explained (Schech et al. 2013; Thurnher et al. 2013). Combination strategies using statins with prenyltransferase inhibitors display synergistic anticancer effects (Konstantinopoulos and Papavassiliou 2007), and likewise, the bisphosphonates show synergism with farnesyl transferase inhibitors (Andela et al. 2002).

Paget’s ‘seed and soil’ theory (Paget 1989) indicates that disseminated cancer cells only fulfil their potential for invasion and proliferation when they are able to adapt to their new environment. Distal metastases are under stress by their new microenvironment and by any systemic drug regimen—the response being either adaptive (Allen and Louise Jones 2011) or quiescent (Redmond et al. 2008). Quiescent cells, including micro-metastases, can remain dormant for years yet changes to the microenvironment can then trigger unrestrained proliferation and the re-emergence of disease (Mimeault and Batra 2010a, b). It is likely that MET enables the cell to re-acquire the signal processing apparatus that allows cognition of the microenvironment (Monteiro and Fodde 2010), and this research explores the hypothesis that both EMT and MET are responsive to mevalonate pathway inhibitors in lung cells in a process that is independent from protein isoprenylation.

EMT can be induced by extrinsic factors such as hepatocyte growth factor, vascular endothelial growth factor, platelet-derived factor and others (Thiery et al. 2009). EMT cells are not only motile but also non-senescing and refractory to apoptosis-inducing treatments. Van Zijl calls this amoeboid phenotype ‘the ultimate exit strategy of cancer cells’ (van Zijl et al. 2011). A switch into MET is needed for the cell to undergo differentiation into the distal organ identity and lose the plasticity needed for invasion. The absence of extracellular matrix (ECM) recognition at this stage can cause the micrometastases to become dormant, and it is proposed that this cognition is inhibited by selective disruption of caveolae, at least in lung cells. The degree that Cav-1 protein is phosphorylated at Tyr14 of the amino terminus increases in response to signals and insulin, EGF, PDGF and Src-family kinases are implicated in this phosphorylation. It is known that depletion of cholesterol causes changes to caveolae formation and localisation, and these changes have been linked to nitric oxide synthase (NOS) activation (Everson and Smart 2001). It has been proposed that caveolae are a scaffold for eNOS as they are co-localised. Over-expression of caveolin-1 has been observed to prevent statins and VEGF from stimulating NOS-mediated angiogenesis (Brouet et al. 2001).

Using four types of inhibitor, this study was designed to reveal the effects of multi-stage blockade of mevalonate and associated pathways on gene expression in two contrasting cancer cell lines. Caveolae have distinctive hydrophilic interior and are entry points to some important growth factors, including hormones, and are thus a critical signalling conduit. This signalling was investigated using array techniques to determine whether caveolae could be specifically inhibited and to assess the effects of such an intervention on EMT.

## Experimental

### Sources

MDA-MB-231 cells were obtained from Cell Lines Service, Eppenheim, Germany, and CaLu-1 cells were obtained from ECCAC, UK. Illumina Human HT12_V4_0_R2_15002873_B human expression microarrays were purchased from Gen-Probe Ltd, UK, and University of Texas Medical School. RNeasy Maxi Kit was purchased from QIAGEN Ltd. All other reagents were sourced from Sigma Aldrich Ltd, UK, except where noted. The antibody array slides and associated reagents were purchased from Full Moon Biosystems Inc, USA.

#### Treatments

Pravastatin (as sodium salt hydrate), a hydrophilic statin, has previously been shown to have pro-apoptotic properties that are dependent upon inhibition of HMG-CoA reductase (Kato et al. 2010); proadifen hydrochloride (N,N-diethylaminoethyl 2, 2-diphenylethanoate hydrochloride) is a Δ-24 dehydrocholesterol reductase (DHCR) inhibitor. Proadifen truncates the pathway at the distal desmosterol and lanosterol intermediates and prevents conversion of 4,4-dimethylcholesta-8 (Thurnher et al. 2013), 24-dien-3β-ol into 4,4-dimethylcholesta-8[9], 24-en-3β-ol, zymosterol into 7-dihydroxyzymesterol, 7,24-cholestadien-3β-ol into 7-cholesten-3β-ol and 5,7,24-cholestatrien-3β-ol into 5,7-cholestadien-3β-ol.

Zoledronic acid monohydrate (Alfa Aesar Chemicals) was used as a typical bisphosphonate, and fatostatin is a specific inhibitor of sterol regulatory element-binding proteins (SREBPs). Zoledronic acid is an aminobisphosphonate prescribed for osteoporosis as it slows down bone reabsorption. It has also been used to suppress prenylation of GTPases and has anti-proliferative and anti-metastatic effects on MDA-MB-231 (Reinholz et al. 2002) and other cells. It has also been shown that zoledronic acid can reverse the EMT through inactivation of NF-κB in MDA-MB-231 (Schech et al. 2013). Fatostatin is a diarylthiazole derivative inhibitor of SREBPs by binding to the escort protein SCAP (Kamisuki et al. 2009).

#### Dose

Each treatment was prepared to the final concentration in fresh media for 24 h. The final concentrations of pravastatin and proadifen in culture flasks were 8.0 µM and 8.5 × 10^−5^ % (w/v), respectively. The dose of pravastatin was chosen because low millimolar serum levels are attainable in vivo at high doses (Wojtkowiak et al. 2011). Zoledronic acid was used at a final concentration of 100 µM, a dose chosen to enable direct comparison with a published data set (Vintonenko et al. 2012). Fatostatin hydrobromide was used at 10 µM after solubilisation in dimethyl sulfoxide in accordance with published work (Li et al. 2014).

#### Gene expression assays

MDA-MB-231 cells were grown to 80 % confluence in Dulbecco’s modified eagles medium (DMEM) supplemented with 10 % v/v foetal bovine serum (FBS), 4500 mg glucose/L 110 mg sodium pyruvate/L and l-glutamine prior to drug exposure. CaLu-1 cells were grown in supplemented minimum essential medium (MEM) with 15 % FBS.

All treatments and controls were conducted in triplicate, and the HT12 v4 Illumina microarray assay was performed using pooled biological replicates. There were no technical replicates except where noted. Cells were treated in 174-ml culture flasks (Nunc A/S) containing the inhibitor dissolved in 40 ml DMEM/MEM with 10 % (v/v) foetal bovine serum (FBS) per treatment. Negative control flasks contained only the FBS-supplemented media. Treatments were 24 h, and treatment start time was 24 h after sub-culture. Incubation was at 37 °C with 5.0 % CO_2_. Approximately 1 × 10^6^ cells from each treatment were harvested with 0.5 g/L porcine trypsin w/v and 0.2 g/L w/v EDTA in Dulbecco phosphate buffer and immediately spun down to a cell pellet. The cells were then re-suspended in phosphate-buffered saline (PBS) pH 7.4 containing 0.1 % of Sigma protease inhibitor cocktail and then re-centrifuged. The resultant cell pellet was then stored in LN_2_ prior to RNA extraction. Array analysis was performed in accordance with the manufacturer’s guidance (http://support.illumina.com/content/dam/illumina-support/documents/myillumina/3466bf71-78bd-4842-8bfc-393a45d11874/wggex_direct_hybridization_assay_guide_11322355_a.pdf).

#### Protein array

Treatment and control solutions each were added directly to three culture flasks at 80 % of confluence for 24 h prior to harvesting. Cells from each triplicate were pooled after the trypsinisation step. Approximately 1 × 10^6^ MDA-MB-231 or CaLu-1 cells were harvested from the flasks using Trypsin–EDTA solution, centrifuged to a pellet and protected with 100 µl of Sigma protease inhibitor cocktail. A signalling explorer array (SET100) comprising 1358 antibodies was used to quantify a range of proteins. The tests were performed in accordance with the manufacturers’ protocols (http://www.fullmoonbio.com/datasheets/AntibodyArrayUserGuide.pdf).

#### Caveolin-1 assay

Cells were gown in 12-well plates (Nunc A/S) until at approximately 50 % confluence. Media in treated wells was replaced with media plus proadifen at a final concentration of 100 µM, and the plates were left for 24 h. The plates were then washed twice in PBS and re-filled with 2 ml per well of Dulbecco’s phosphate buffer containing anti-caveolin-1 conjugated to fluorescein isothiocyanate (FITC). The plates were incubated for a further 2 h, washed repeatedly with PBS and then scanned in a fluorescence plate reader (FL500 BioTek Instruments Inc.).

#### Statistical treatment

Gene expression raw array data were assessed for quality, and outliers removed. Raw data were deposited at the NIH Geo database (see Table 1). A total of 47,319 features were transformed using a variance stabilising transformation method prior to normalisation across all arrays using the robust spline normalisation method. Data were filtered to remove data with *p* values ≥ 0.05, and a further filter was applied to remove all genes not within the EMT subset (the gene subset was compiled using the Qiagen Profiler PCR array EMT gene list and augmented with genes associated with rafts and caveolae. (SI Table 1). Mean expression measures (summarised intensities) are in log base 2. The protein assay was performed with technical replicates using pooled cells from three replicate flasks. Array data with a coefficient of variation <0.1 were removed.

**Table 1:** Sample numbers for the treatments indicate biological replicates

Treatment	MDA-MB-231	CaLu-1
Pravastatin	**4**	**3**
	GSE47461	GSE47458
Proadifen	**4**	**3**
	GSE47461	GSE47458
Zoledronic acid	4	**2**
	GSM829774	GSE70027
	GSM829775	
	GSM829776	
	GSM829777	
	GSM829810	
	GSM829811	
	GSM829812	
	GSM829813	
Fatostatin	2	**2**
	GSE70027	GSE70027
Fatostatin and Proadifen	2	**2**
	GSE70027	GSE70027
Control	**4**	**3**
	GSE47461	GSE47458

www.ncbi.nlm.nih.gov/geo/

**Fig. 1:**
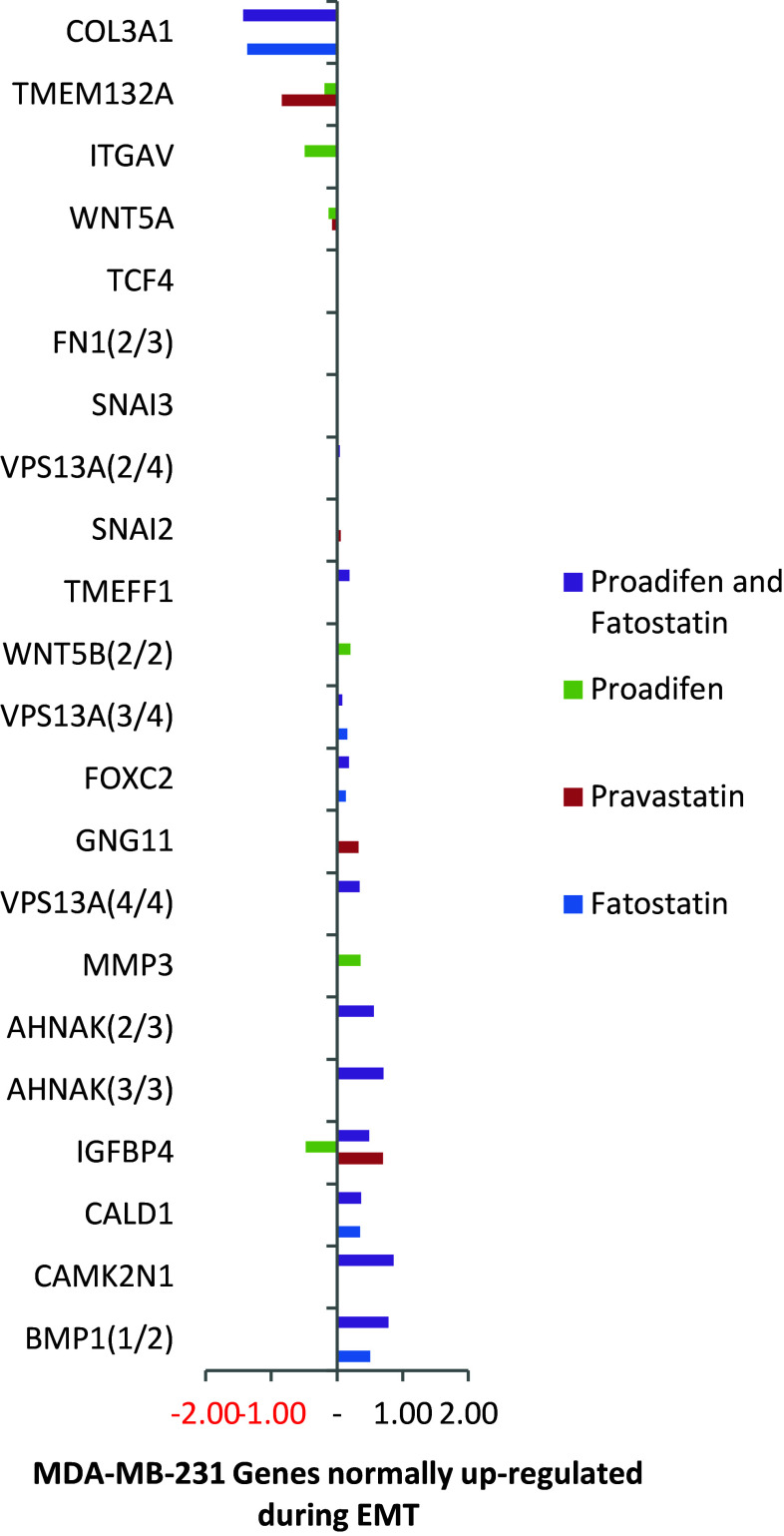
The effects of the treatments on MDA-MB-231 cells. The data (*p* < 0.05) are confined to a subset of genes classically up-regulated during EMT. Proadifen and to a lesser degree pravastatin cause some significant movements, notably COL3A1 that codes for collagen type III alpha 1. Zoledronic acid caused no effects at *p* < 0.05 in this Ras-negative cell line

**Fig. 2:**
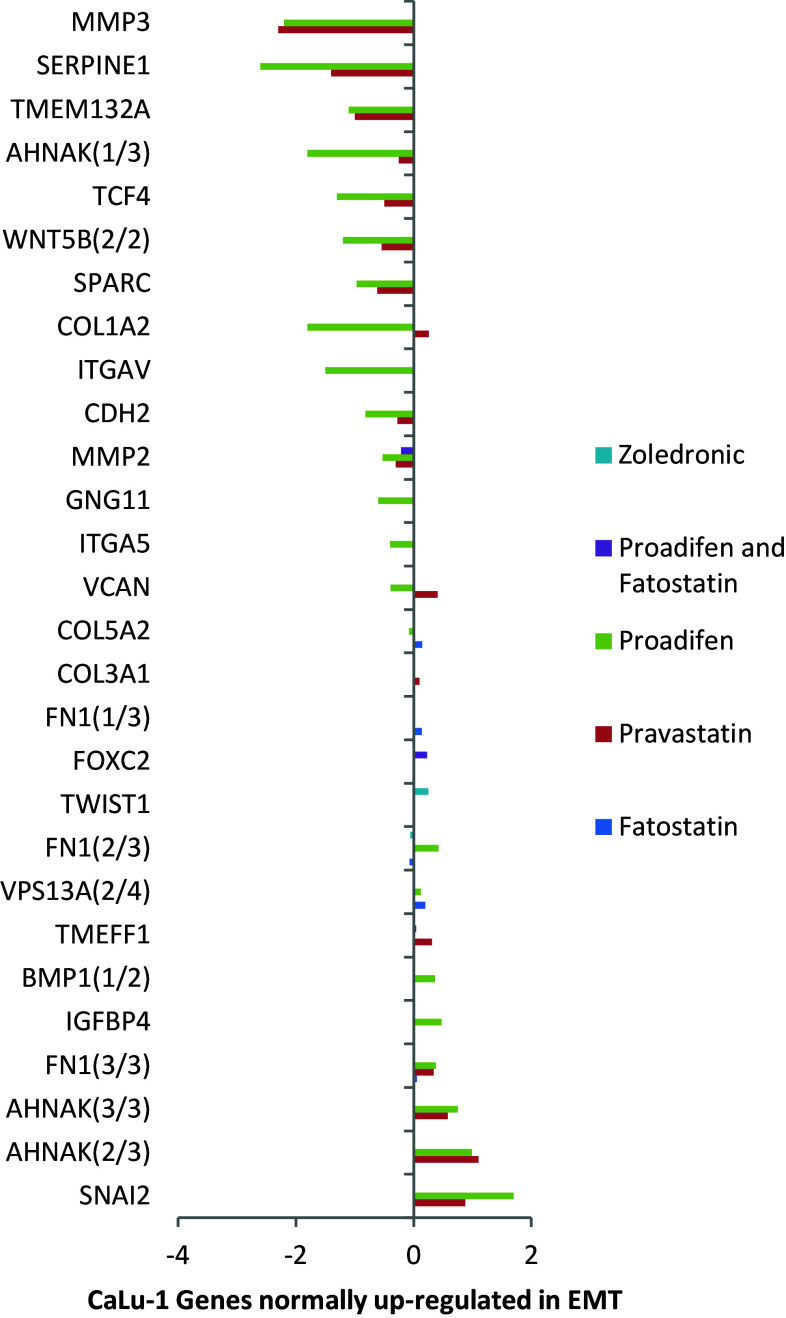
The response to the same treatments in CaLu-1 cells where significant changes to gene expression can be seen. Zoledronic acid displays a modest effect in FOXC2 (transcription factor FKH-14 associated with EMT) and FN1 (a fibronectin)

**Fig. 3:**
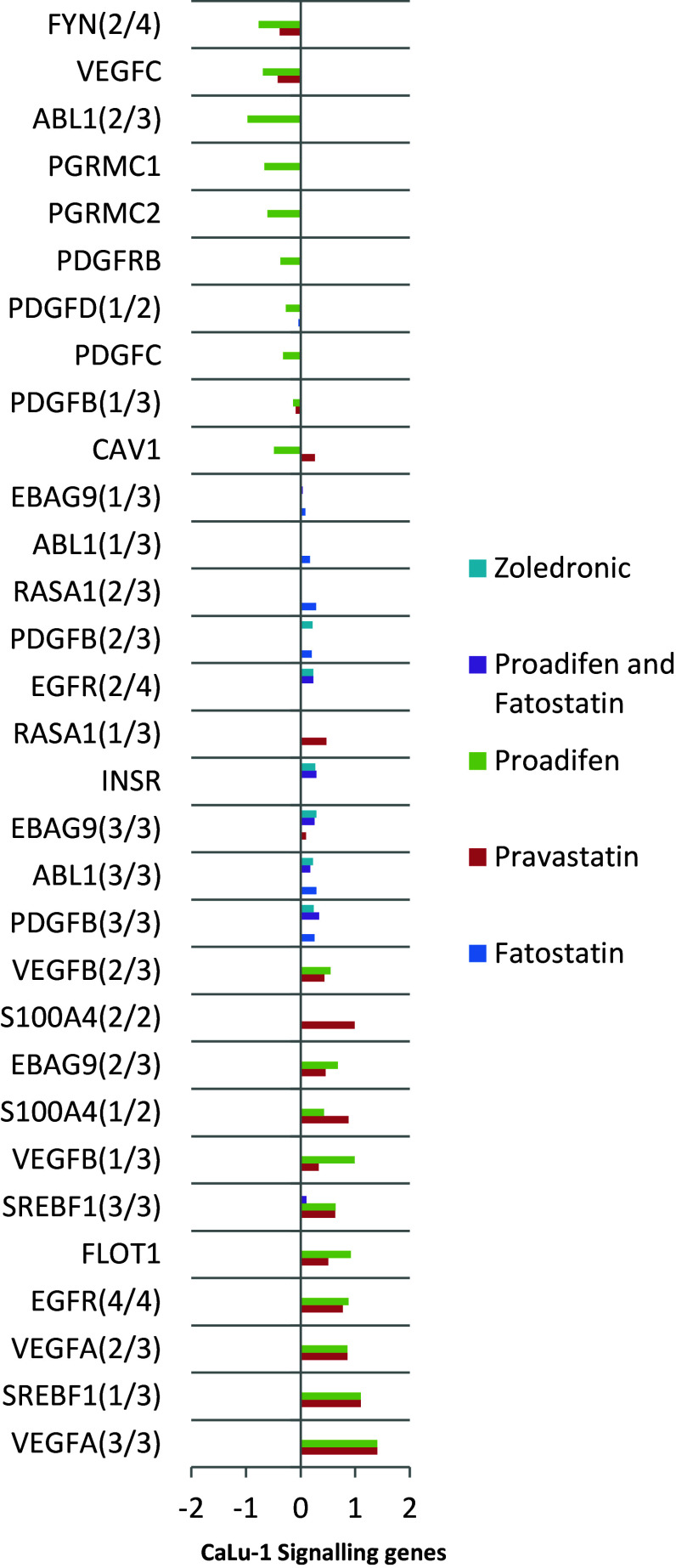
The effects of the treatments on CaLu-1 cells. The data are filtered for significance (*p* < 0.05) and by the subset of genes associated with signalling. X-axis shows log2 fold change. Transcription of FYN and ABL1 is reduced; both FYN and ABL proteins mediate transduction at caveolae. PDGF and PDGF/R are likewise down-regulated. The down-regulated genes at the top of the graph are associated with caveolae, while the up-regulated genes at the lower part of the graph are associated with rafts

**Fig. 4:**
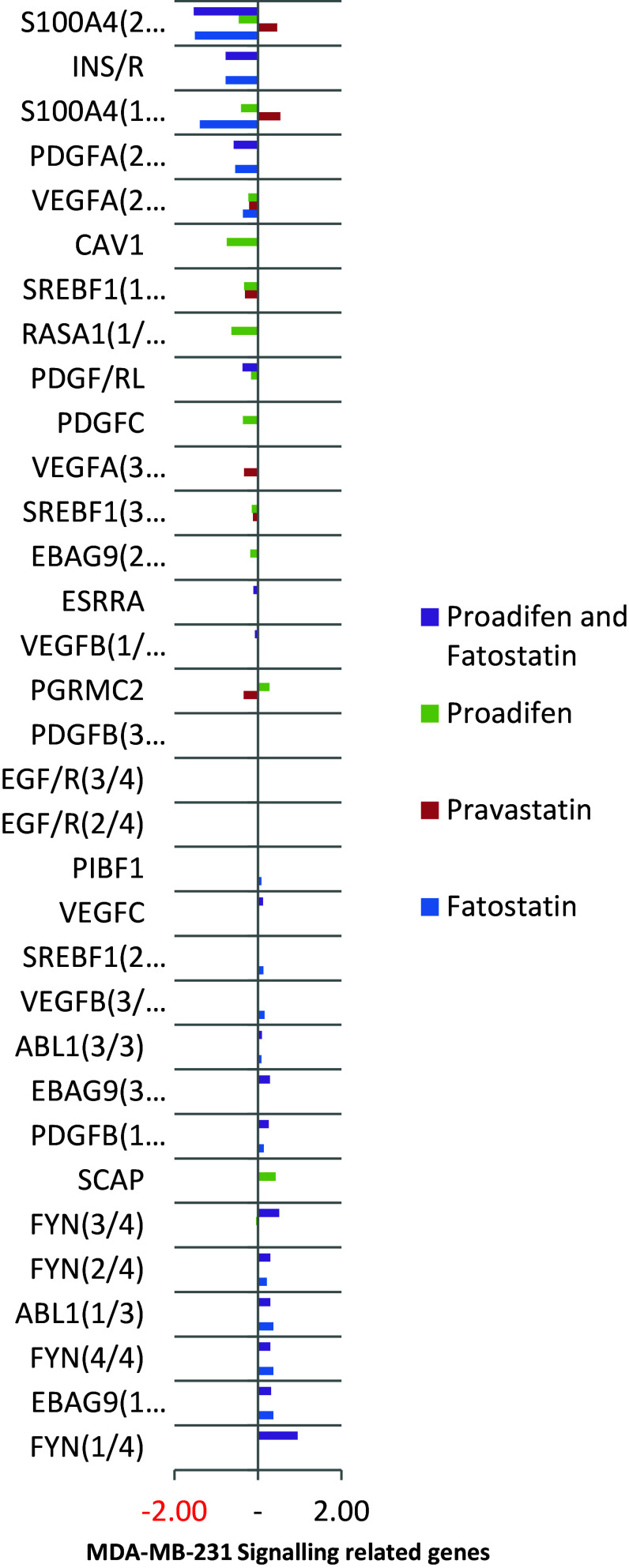
FYN and ABL1 are up-regulated by fatostatin, while proadifen causes an up-regulation of SCAP, a protein associated with caveolae. CAV1 and RASA1 are down-regulated by proadifen. Splice variants are numbered x/y, and their identifiers are listed in the supplementary data

mRNA array data were confirmed by RT-qPCR. Cross-validation data were available for 70 % of the entire subset using QIAGEN human VEGF signalling array (PAHS-091Z) and MET array (PAHS-090Z) in combination for proadifen and pravastatin treatments in both cell lines (8 samples). This additional data showed that in accordance with recent published reviews of validation metadata (Wang et al. 2006) there was a high overall correlation between the RNA microarray and qRT-PCR results. Subset genes appearing in Figs. 1, 2, 3 and 4 are extracted, and corresponding PCR data are presented in SI Table 3.

## Results

**Fig. 5:**
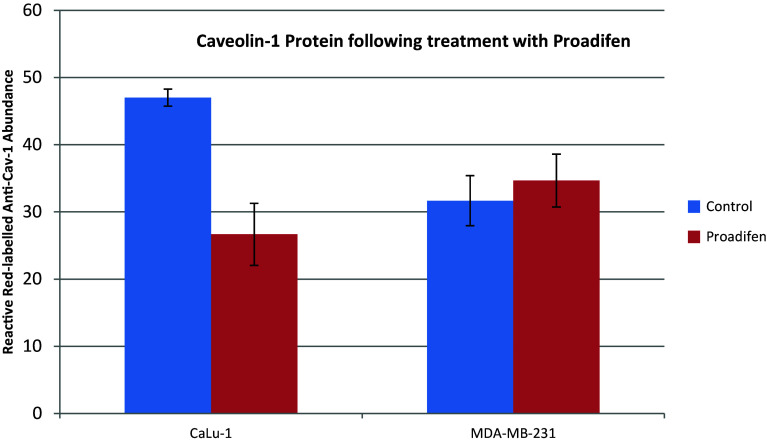
*CAV1* down-regulation is mirrored in the CAV-1 protein assay only in CaLu-1 cells. Expressed protein levels in MDA-MB-231 cells are not significantly different from the control groups. *Error bars* are ±SE

**Fig. 6:**
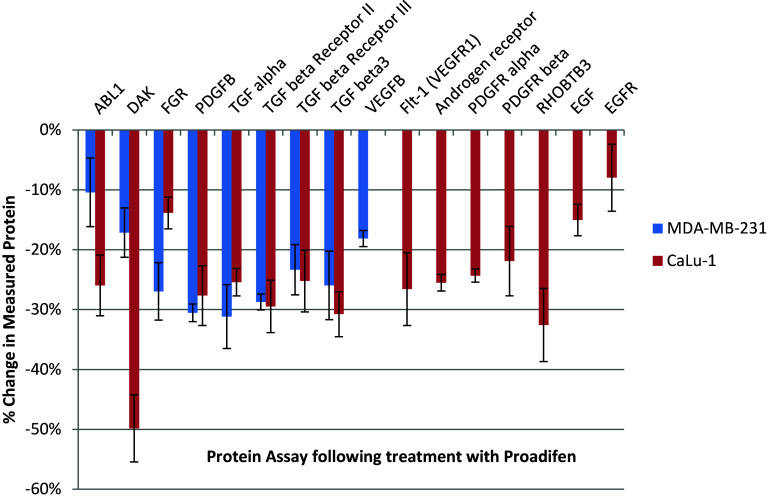
MDA-MB-231 cells share a reduction in half of the signal proteins compared to CaLu-1. In only the lung cells the receptors for EGF, PDGF, androgens and VEGF are affected by the treatment. *Error bars* are ±SE

Within the subset of genes normally up-regulated during EMT in MDA-MB-231, there were 22 significant up-/down-regulations between −1.75 and +0.5 log2 fold change (FC), and the majority of these genes were up-regulated (Fig. 1). In contrast, CaLu-1 cells showed greater sensitivity to the inhibitors with 28 affected genes showing responses that ranged from −2.60 to +1.7 log2 FC (Fig. 2). Proadifen had the greatest effect, shadowed by the statin treatment. Zoledronic acid and fatostatin had little effect in CaLu-1 cells but did have some effect on the breast line. Within the subset of genes related to signalling, both cells responded to the inhibitors with the CaLu-1 line showing clear effects on regulation of caveolae-associated proteins such as the non-receptor kinases *FYN* and *ABL*. Pravastatin generally tracked but lagged proadifen in CaLu-1. MDA-MB-231 was largely unresponsive to the statin and proadifen. *CAV1* was down-regulated in both lines by proadifen with this down-regulation being translated into protein expression only in CaLu-1 cells (Fig. 5). The protein array analyses showed only down-regulations in both cell lines with 7 proteins only impacted by proadifen in CaLu-1. Significantly, these include the receptors for EGF, VEGF, PDGF and androgens (Fig. 6).

## Discussion and conclusions

Much research effort has been focussed on the reduction in cancer-related morbidity and mortality in long-term statin users (Hindler et al. 2006). Statins competitively inhibit the HMG-CoA reductase enzyme found at the beginning of the mevalonate pathway. They have been implicated in pleiotropic effects such as inflammation, immune modulation and autophagy of cancer cells, while epidemiological studies suggest a beneficial role in cancer recurrence and it has been reported previously that gene expression of cancer cells treated with statins in vitro is profoundly altered (Garnett and Greenhough 2012). The mevalonate pathway intermediates geranylgeranyl pyrophosphate (GGPP) and farnesylpyrophosphate (FPP) are responsible for the isoprenylation of numerous GTPase signal proteins, most notably the RhoA superfamily. The signal transduction of farnesylated Ras (Casey et al. 1989) and Rho is blocked by farnesyl transferase inhibitors that have anticancer effects (Oliff 1999).

In CaLu-1, where there was a detectable effect of zoledronic acid, there was no overlap in gene response with the other treatments, suggesting that a double blockade of isoprenylation and Δ-24 oxidoreductase would have the greatest impact on cellular status. Zoledronic acid had no effect on the transcription of genes within the EMT subset in MDA-MB-231, and this is likely to be due to the Ras-inactive status of this cell line.

SREBP feedback to the transcriptome is truncated by fatostatin, and additional cholesterol is not manufactured and deployed to the construction of invadopodia or the caveolae superstructure. Likewise, inhibition of free cholesterol per se by proadifen or pravastatin will disable the cell from forming this new architecture. The combination of fatostatin and proadifen is marginally synergistic. A perturbation of rafts in MDA-MB-231 is sufficient to inhibit EGF/R signalling (Rogers et al. 2010). Caveolae have a specific requirement for free cholesterol to intercalate with Cav-1 (Murata et al. 1995), while rafts appear able to form with cholesterol intermediates and remain functional. This is supported by gene knockout mice (DHCR24^−/−^) that cannot complete the Bloch pathway yet remain viable (Wechsler et al. 2003) utilising the precursor desmosterol for structural and ligand-binding purposes. This suggests that a reduction in free cholesterol will adversely affect caveolae-based signalling to a greater degree than raft signalling.

The MDA-MB-231 line is Ras-inactive and ER-negative and consequently less vulnerable to inhibition of both isoprenylation and hormone stimulation. The results presented here reflect this status, with Pravastatin and zoledronic acid having modest or zero effects, respectively, in this cell line. The role of caveolae in lung cancer cells is revealed: the mRNA data show a significant down-regulation of both *PDGF* and its receptor *PDGF/R*. This is concurrent with a significant reduction in caveolae scaffold *CAV1* and associated trafficking proteins *FYN*, *ABL* and *SCAP* that mediate the ECM signals once the bilayer has been traversed. Down-regulation of *ABL* is not subject to losses in translation, and ABL protein is reduced in both cell lines and very significantly in CaLu-1 (−50 %). Other indicators of pluripotency are affected by the treatments, and the response is cell type specific, with CaLu-1 displaying more sensitivity to the statin and to proadifen compared to MDA-MB-2312. Protein analysis shows that the directionality of the gene regulations is indeed translated to protein expression for some signalling molecules. In CaLu-1 these specifically overlap with a number of growth factors including PDGF, VEGF and EGF. PDGF, PDGF/R (Tiesman and Hart 1993) and VEGF (Kendall et al. 1996) are water soluble and thus may be of particular importance in caveolae-mediated signalling in lung cancer (see Figs. 1, 6) where these signals use caveolae to access the cell. The treatments also caused a reduction in expressed matrix metalloproteinases (FC: MDA-MB-231 ↓0.784; FC: CaLu-1 ↓0.704; SI Table 2), showing that that a reduction in cholesterol in both cell lines caused a reduction in the metastatic phenotype.

Δ-24 DHCR-inhibiting drugs selectively reduce the formation of caveolae, and the consequent deprivation of those extracellular signals that depend on caveolae for transduction causes gene expression to be moved towards quiescence. It is possible that those cells forced to maintain a more epithelial identity may be unable to re-acquire malignant proliferation (Iiizumi et al. 2008; Aguirre-Ghiso 2007).

Normally, as the cell produces invadopodia, signalling associated with invadopodia-localised caveolae will increase; as this occurs there may be an autoinductive response by the cell-promoting invadopodia. Such a cycle would only reinforce EMT, while a disruption in this process would serve to promote quiescence. This research supports the theory that caveolae are a critical conduit for autocrine/paracrine signals that promote the metastatic phenotype and that their abolition is beneficial to cellular identity.

Proadifen inhibits several of the cytochrome p450 mono-oxygenase reactions in a heterogeneous manner (Anders and Mannering 1966), but a more specific Δ-24 inhibitor may have clinical value in the forced dormancy of disseminated tumour cells.

## Electronic supplementary material

Below is the link to the electronic supplementary material.

Supplementary material 1 (DOCX 24 kb)
