# Supplementary material for: Caveolae as a target to quench autoinduction of the metastatic phenotype in lung cancer
Source: J Cancer Res Clin Oncol. 2015 Nov 16;142(3):611–8. doi: 10.1007/s00432-015-2074-3 (PMC4751176; doi:10.1007/s00432-015-2074-3)
Supplement: Supplementary file 1 — Supplementary material 1 (DOCX 24 kb) [file 432_2015_2074_MOESM1_ESM.docx]

| **Table S1** Gene variant symbol list with Illumin ID | | | | | | | | | | | | | |  |  |
| --- | --- | --- | --- | --- | --- | --- | --- | --- | --- | --- | --- | --- | --- | --- | --- |
| U_SYMBOL | | Illumin ID | | | | U_SYMBOL | | | Illumin ID | | | | |  |  |
| ABL1(1/3) | | ILMN_1708922 | | | | MMP3 | | | ILMN_1784459 | | | | |  |  |
| ABL1(2/3) | | ILMN_1713732 | | | | PDGFB(2/3) | | | ILMN_1775822 | | | | |  |  |
| ABL1(3/3) | | ILMN_2268241 | | | | PDGFB(3/3) | | | ILMN_2394305 | | | | |  |  |
| AHNAK(1/3) | | ILMN_1714567 | | | | PDGFRL | | | ILMN_1680339 | | | | |  |  |
| AHNAK(2/3) | | ILMN_1752159 | | | | PGRMC1 | | | ILMN_1684771 | | | | |  |  |
| AHNAK(3/3) | | ILMN_1792495 | | | | PGRMC2 | | | ILMN_2195236 | | | | |  |  |
| BMP1(1/2) | | ILMN_1800412 | | | | PIBF1 | | | ILMN_2227473 | | | | |  |  |
| CALD1 | | ILMN_1717990 | | | | RASA1(1/3) | | | ILMN_1725312 | | | | |  |  |
| CAMK2N1 | | ILMN_1794863 | | | | RASA1(2/3) | | | ILMN_1737576 | | | | |  |  |
| CAV1 | | ILMN_2149226 | | | | S100A4(1/2) | | | ILMN_1684306 | | | | |  |  |
| CDH2 | | ILMN_1779228 | | | | S100A4(2/2) | | | ILMN_1688780 | | | | |  |  |
| COL1A2 | | ILMN_2104356 | | | | SCAP | | | ILMN_1677534 | | | | |  |  |
| COL3A1 | | ILMN_1773079 | | | | SERPINE1 | | | ILMN_1744381 | | | | |  |  |
| COL5A2 | | ILMN_1729117 | | | | SNAI2 | | | ILMN_2082585 | | | | |  |  |
| EBAG9(1/3) | | ILMN_1729144 | | | | SNAI3 | | | ILMN_1690262 | | | | |  |  |
| EBAG9(2/3) | | ILMN_1791896 | | | | SPARC | | | ILMN_1796734 | | | | |  |  |
| EBAG9(3/3) | | ILMN_2273683 | | | | SREBF1(1/3) | | | ILMN_1663035 | | | | |  |  |
| EGFR(2/4) | | ILMN_1728858 | | | | SREBF1(2/3) | | | ILMN_1695378 | | | | |  |  |
| EGFR(3/4) | | ILMN_1755535 | | | | SREBF1(3/3) | | | ILMN_2328986 | | | | |  |  |
| EGFR(4/4) | | ILMN_1798975 | | | | TCF4 | | | ILMN_1814194 | | | | |  |  |
| ESRRA | | ILMN_1774272 | | | | TMEFF1 | | | ILMN_1729498 | | | | |  |  |
| FLOT1 | | ILMN_1661439 | | | | TMEM132A | | | ILMN_2317923 | | | | |  |  |
| FN1(1/3) | | ILMN_1675646 | | | | TWIST1 | | | ILMN_1672908 | | | | |  |  |
| FN1(2/3) | | ILMN_1778237 | | | | VCAN | | | ILMN_1687301 | | | | |  |  |
| FN1(3/3) | | ILMN_2366463 | | | | VEGFA(2/3) | | | ILMN_1803882 | | | | |  |  |
| FOXC2 | | ILMN_1705201 | | | | VEGFA(3/3) | | | ILMN_2375879 | | | | |  |  |
| FYN(1/4) | | ILMN_1686555 | | | | VEGFB(1/3) | | | ILMN_1722855 | | | | |  |  |
| FYN(2/4) | | ILMN_1781207 | | | | VEGFB(2/3) | | | ILMN_1726981 | | | | |  |  |
| FYN(3/4) | | ILMN_2249920 | | | | VEGFB(3/3) | | | ILMN_1801814 | | | | |  |  |
| FYN(4/4) | | ILMN_2380801 | | | | VEGFC | | | ILMN_1701204 | | | | |  |  |
| GNG11 | | ILMN_1782419 | | | | VPS13A(2/4) | | | ILMN_1789828 | | | | |  |  |
| IGFBP4 | | ILMN_1665865 | | | | VPS13A(3/4) | | | ILMN_1800721 | | | | |  |  |
| INSR | | ILMN_1670918 | | | | VPS13A(4/4) | | | ILMN_2414826 | | | | |  |  |
| ITGA5 | | ILMN_1792679 | | | | WNT5A | | | ILMN_1800317 | | | | |  |  |
| ITGAV | | ILMN_2169439 | | | | WNT5B(2/2) | | | ILMN_1772824 | | | | |  |  |
| **Table S2** | | Fold changes in MMP protein levels in MDA-MB-231 and CaLu-1 cells following treatment with Proadifen | | | | | | | | | | | | | |
|  | | Fold change data points where CV=0 or ≥0.1 have been removed | | | | | | | | | | | | | |
| Matrix  Metalloproteinases | | Fold Change | | | Average Signal of Replicate Spots on the Array | | | | | | Coefficient of Variation for Replicate Spots | | | | |
|  | | MDA-MB231 | | CaLu-1 | MDA-MB-231 Treated | MDA-MB-231 Control | | CaLu-1 Treated | CaLu-1 Control | | MDA-MB-231 Treated | MDA-MB-231 Control | CaLu-1 Treated | CaLu-1 Control | |
| MMP1 (Cleaved-Phe100) | |  | |  | 270 | 190 | | 335 | 241 | | 0.01 | 0.08 | 0.02 | 0 | |
| MMP-10 | |  | |  | 2895 | 2088 | | 3471 | 2548 | | 0.01 | 0.03 | 0.02 | 0.03 | |
| MMP-11 | | 0.757 | | 0.674 | 696 | 527 | | 881 | 594 | | 0.06 | 0 | 0 | 0.02 | |
| MMP12 (Cleaved-Glu106) | | 0.721 | | 0.772 | 330 | 238 | | 408 | 315 | | 0.01 | 0.18 | 0.04 | 0.01 | |
| MMP-13 | |  | |  | 590 | 493 | | 790 | 538 | | 0.06 | 0.01 | 0 | 0.02 | |
| MMP-14 | | 0.865 | | 0.672 | 385 | 333 | | 467 | 314 | | 0.03 | 0.07 | 0.02 | 0.01 | |
| MMP14 (Cleaved-Tyr112) | |  | |  | 503 | 364 | | 561 | 509 | | 0.08 | 0.02 | 0.28 | 0 | |
| MMP-15 | |  | |  | 451 | 317 | | 562 | 332 | | 0.02 | 0.17 | 0 | 0.01 | |
| MMP15 (Cleaved-Tyr132) | | 0.907 | | 0.792 | 313 | 284 | | 355 | 281 | | 0.02 | 0.03 | 0.03 | 0.1 | |
| MMP-16 | |  | |  | 425 | 292 | | 475 | 320 | | 0.03 | 0.15 | 0.03 | 0 | |
| MMP17 (Cleaved-Gln129) | |  | |  | 374 | 264 | | 441 | 339 | | 0 | 0.08 | 0.03 | 0.11 | |
| MMP-19 | | 0.735 | | 0.659 | 682 | 501 | | 778 | 513 | | 0.08 | 0.13 | 0.09 | 0.02 | |
| MMP-2 | |  | |  | 1268 | 1135 | | 1505 | 1242 | | 0.25 | 0.01 | 0.04 | 0.22 | |
| MMP-23 | |  | |  | 351 | 248 | | 410 | 295 | | 0.01 | 0.11 | 0.04 | 0 | |
| MMP23 (Cleaved-Tyr79) | |  | |  | 3319 | 1588 | | 1731 | 2278 | | 0.01 | 0.27 | 0.04 | 0.13 | |
| MMP27 (Cleaved-Tyr99) | |  | |  | 843 | 510 | | 1024 | 662 | | 0.15 | 0.09 | 0.17 | 0.07 | |
| MMP-3 | |  | |  | 415 | 292 | | 531 | 353 | | 0.11 | 0.15 | 0.14 | 0.05 | |
| MMP3 (Cleaved-Phe100) | |  | |  | 369 | 309 | | 451 | 342 | | 0.07 | 0.05 | 0 | 0.05 | |
| MMP-7 | |  | |  | 570 | 378 | | 634 | 467 | | 0.04 | 0.21 | 0.14 | 0.08 | |
| MMP-8 | | 0.72 | | 0.68 | 564 | 406 | | 699 | 475 | | 0.01 | 0.13 | 0.04 | 0.09 | |
| MMP-9 | | 0.789 | | 0.684 | 332 | 262 | | 393 | 269 | | 0.01 | 0.04 | 0.03 | 0.01 | |

| **Table S3** qRT-PCR Data for Gene Subset reported in Figs 1-4 | | | |  |  |  |  |  |  |  |
| --- | --- | --- | --- | --- | --- | --- | --- | --- | --- | --- |
| CALU-1 Signalling | | | | |  | CALU-1 Genes Up-regulated during EMT | | | | |
| Gene ID | Pravastatin Ct | CV% | Proadifen Ct | CV |  | Gene ID | Pravastatin Ct | CV | Proadifen Ct | CV |
| VEGFA | 0.0111 | 1.6890 | 0.0255 | 1.3270 |  | AHNAK | 0.0705 | 0.7364 | -0.0017 | 1.0670 |
| EGFR | 0.0067 | 0.0674 | 0.0105 | 0.8098 |  | MMP3 | -0.0436 | 0.9710 | 0.0776 | 1.0680 |
| VEGFB | 0.0130 | 2.7440 | -0.0037 | 2.5630 |  | FN1 | 0.0304 | 0.1954 | 0.0005 | 0.9614 |
| PDGFC |  |  | -0.0509 | 2.0960 |  | COL1A2 | 0.0859 | 0.8690 | -0.0648 | 7.7120 |
| VEGFC | -0.0109 | 0.4440 | -0.0503 | 3.0170 |  | BMP1 |  |  | -0.0006 | 0.1766 |
| PDGFRB |  |  | -0.0925 | 4.0280 |  | COL5A2 |  |  | -0.0038 | 1.0980 |
| CAV1 | -0.0521 | 2.9200 | -0.1874 | 2.6550 |  | GNG11 |  |  | -0.0107 | 1.5300 |
|  |  |  |  |  |  | ITGA5 |  |  | -0.0110 | 0.9996 |
| MB-231 Signalling | | | | |  | TMEFF1 | -0.0159 | 0.9820 |  |  |
| Gene ID | Pravastatin Ct | CV | Proadifen Ct | CV |  | VPS13A |  |  | -0.0335 | 2.0700 |
| VEGFA | 0.0675 | 3.0540 | -0.1023 | 4.3810 |  | MMP2 | 0.0113 | 1.6050 | -0.0481 | 2.4180 |
| EGFR |  |  | -0.1225 | 4.7760 |  | CDH2 | -0.0074 | 0.6808 | -0.0308 | 3.7650 |
| PDGFC |  |  | -0.1247 | 2.9350 |  | SNAI2 | -0.0013 | 1.3040 | -0.0410 | 1.7090 |
| CAV1 |  |  | -0.1376 | 3.6880 |  | ITGAV |  |  | -0.0441 | 0.7830 |
|  |  |  |  |  |  | COL3A1 | -0.0571 | 0.8900 |  |  |
| MB-231 Genes Up-regulated during EMT | | | | |  | TMEM132A | -0.0521 | 1.5020 | -0.0226 | 3.6610 |
| Gene ID | Pravastatin Ct | CV | Proadifen Ct | CV |  | IGFBP4 |  |  | -0.0770 | 1.4490 |
| SNAI2 | 0.0679 | 0.7573 |  |  |  | SPARC | -0.0073 | 2.3060 | -0.1395 | 3.5060 |
| GNG11 | -0.0126 | 5.1522 |  |  |  | SERPINE1 | -0.0271 | 2.1570 | -0.1248 | 0.3866 |
| MMP3 |  |  | -0.0170 | 0.1654 |  | TCF4 | -0.0461 | 0.6817 | -0.1063 | 0.2640 |
| WNT5A | -0.0380 | 0.5829 |  |  |  | VCAN | 0.0121 | 3.7380 | -0.1875 | 1.6627 |
| TMEM132A | 0.0411 | 8.2590 | -0.1077 | 4.1534 |  |  |  |  |  |  |
| FN1 |  |  | -0.1033 | 4.8460 |  |  |  |  |  |  |
| ITGAV |  |  | -0.1201 | 4.9540 |  | Fold change data points where CV=0 or ≥0.1 have been removed | | | | |
| IGFBP4 | -0.0260 | 6.3261 | -0.1520 | 2.3270 |  |  |  |  |  |  |
